# Di-μ-iodido-bis{[hydroxy(methoxy)bis(2-pyridyl)methane-κ^3^ *N*,*O*,*N*′]iodidocadmium(II)}

**DOI:** 10.1107/S1600536810041681

**Published:** 2010-10-23

**Authors:** Majid Esmhosseini, Nasser Safari, Vahid Amani

**Affiliations:** a Department of Chemistry, University of Urmiyeh, Urmyieh, Iran; b Department of Chemistry, Shahid Beheshti University, G. C., Evin, Tehran 1983963113, Iran

## Abstract

In the centrosymmetric dinuclear title compound, [Cd_2_I_4_(C_12_H_12_N_2_O_2_)_2_], two μ-I atoms bridge two Cd^II^ atoms and each Cd^II^ atom is also bonded to a terminal I atom and a hy­droxy-meth­oxy-bis­(2-pyrid­yl)methane ligand, which functions in an *N*,*O*,*N*′-tridentate mode, resulting in a distorted octa­hedral coordination environment. Inter­molecular O—H⋯I hydrogen bonds and π–π stacking inter­actions between the pyridine rings [centroid–centroid distance = 3.790 (2) Å] are present in the crystal structure.

## Related literature

For general background to metal complexes with bis­(2-pyrid­yl)ketone or derivative ligands, see: Bandoli *et al.* (1994 ▶); Breeze *et al.* (1996 ▶); Crowder *et al.* (2004 ▶); Hemmert *et al.* (1999 ▶); Katsoulakou *et al.* (2002 ▶); Kavounis *et al.* (1996 ▶); Padhi & Sahu (2008 ▶); Papadopoulos *et al.* (1996 ▶); Rattanaphani & McWhinnie (1974 ▶); Serna *et al.* (2001 ▶); Sommerer *et al.* (1993 ▶); Tangoulis *et al.* (1997 ▶).
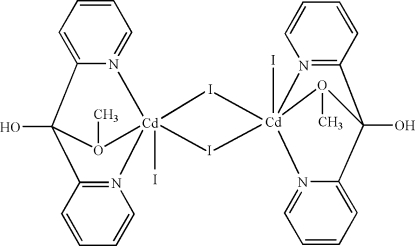


## Experimental

### 

#### Crystal data

[Cd_2_I_4_(C_12_H_12_N_2_O_2_)_2_]
*M* *_r_* = 1164.89
Monoclinic, 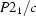

*a* = 9.6684 (6) Å
*b* = 10.1083 (7) Å
*c* = 16.4970 (13) Å
β = 105.365 (5)°
*V* = 1554.64 (19) Å^3^
*Z* = 2
Mo *K*α radiation
μ = 5.38 mm^−1^
*T* = 298 K
0.33 × 0.09 × 0.05 mm

#### Data collection

Bruker APEX CCD diffractometer
Absorption correction: multi-scan (*SADABS*; Sheldrick, 1996 ▶) *T*_min_ = 0.560, *T*_max_ = 0.760
12218 measured reflections
4186 independent reflections
3589 reflections with *I* > 2σ(*I*)
*R*_int_ = 0.037

#### Refinement

*R*[*F* ^2^ > 2σ(*F* ^2^)] = 0.027
*wR*(*F* ^2^) = 0.056
*S* = 1.08
4186 reflections
174 parameters
H-atom parameters constrained
Δρ_max_ = 0.61 e Å^−3^
Δρ_min_ = −0.69 e Å^−3^

### 

Data collection: *SMART* (Bruker, 2007 ▶); cell refinement: *SAINT* (Bruker, 2007 ▶); data reduction: *SAINT*; program(s) used to solve structure: *SHELXTL* (Sheldrick, 2008 ▶); program(s) used to refine structure: *SHELXTL*; molecular graphics: *ORTEP-3* (Farrugia, 1997 ▶); software used to prepare material for publication: *WinGX* (Farrugia, 1999 ▶).

## Supplementary Material

Crystal structure: contains datablocks I, global. DOI: 10.1107/S1600536810041681/hy2362sup1.cif

Structure factors: contains datablocks I. DOI: 10.1107/S1600536810041681/hy2362Isup2.hkl

Additional supplementary materials:  crystallographic information; 3D view; checkCIF report

## Figures and Tables

**Table 1:** Selected bond lengths (Å)

Cd1—N1	2.381 (3)
Cd1—N2	2.371 (3)
Cd1—O1	2.633 (2)
Cd1—I1	2.8868 (4)
Cd1—I1^i^	2.9951 (4)
Cd1—I2	2.8082 (4)

Symmetry code: (i) 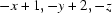.

**Table 2:** Hydrogen-bond geometry (Å, °)

*D*—H⋯*A*	*D*—H	H⋯*A*	*D*⋯*A*	*D*—H⋯*A*
O2—H2*A*⋯I2^ii^	0.82	2.73	3.509 (3)	160

Symmetry code: (ii) 
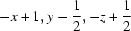
.

**Fig. 1.**
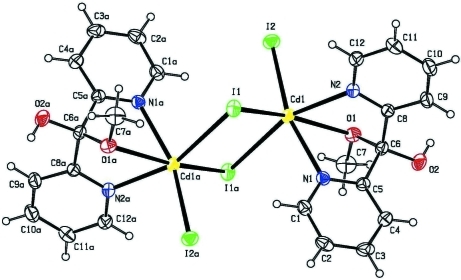
The molecular structure of the title compound. Displacement ellipsoids are drawn at the 50% probability level. [Symmetry code: (a) 1-x, 2-y, -z.]

**Fig. 2.**
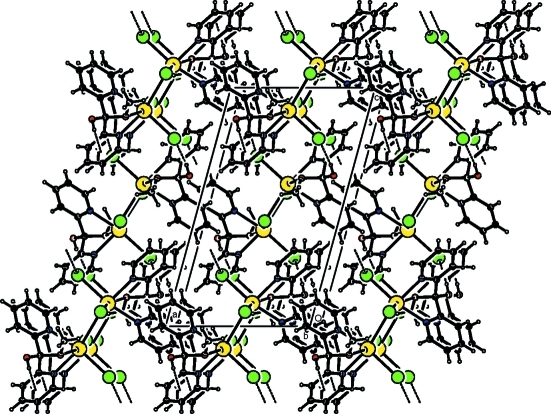
Packing diagram of the title compound. Dashed lines denote hydrogen bonds.

## supplementary crystallographic information

### Comment

Bis(2-pyridyl)ketone (bpk) is a molecule that can exhibit different modes of coordination. As a bidentate ligand, it may present an N,O-coordination giving a five-membered chelate ring or an N,N-coordination forming a three or six-membered ring (Crowder *et al.*, 2004; Rattanaphani & McWhinnie, 1974; Sommerer *et al.*, 1993). Frequently the coordinated bpk undergoes nucleophilic addition of water or an alcohol at the carbonylic carbon atom to form the diol or the corresponding hemiacetal, (pyridyl)_2_C(OR)(OH), which, deprotonated, acts as a mononegative, tridentate N,O,N-donor ligand. The three donor atoms may be coordinated to one metal atom (Bandoli *et al.*, 1994; Crowder *et al.*, 2004; Kavounis *et al.*, 1996; Padhi & Sahu, 2008) or to two metal atoms in a bridging coordination (Breeze *et al.*, 1996; Hemmert *et al.*, 1999; Katsoulakou *et al.*, 2002; Papadopoulos *et al.*, 1996; Serna *et al.*, 2001; Tangoulis *et al.*, 1997). Here, we report the synthesis and structure of the title compound.

The asymmetric unit of the title compound contains a half of the molecule (Fig. 1). Two µ-I atoms bridge two Cd^II^ atoms, and each Cd^II^ atom is also bonded to a terminal I atom and an organic ligand which functions in an N,O,N'-tridentate mode, resulting in a distorted octahedral coordination environment. The Cd—I, Cd—O and Cd—N bond lengths are collected in Table 1.

In the crystal structure, intermolecular O—H···I hydrogen bonds (Table 2) and π–π interactions between the pyridine rings (Fig. 2), *Cg*···*Cg*^i^ [symmetry code: (i) -*x*, 2-y,-*z*; *Cg* is the centroid of the N1, C1–C5 ring], stabilize the structure, with a centroid-centroid distance of 3.790 (2) Å.

### Experimental

For the preparation of the title compound, a solution of bis(2-pyridyl)ketone (0.15 g, 0.80 mmol) in methanol (10 ml) was added to a solution of CdI_2_ (0.29 g, 0.80 mmol) in methanol (5 ml) at room temperature. Crystals suitable for X-ray diffraction experiment were obtained by methanol diffusion into a colorless solution in DMSO. The crystals were isolated after one week (yield: 0.34 g, 72.9%).

### Refinement

H atoms were positioned geometrically and refined as riding atoms, with C—H = 0.93 (aromatic) and 0.96 (methyl), O—H = 0.82 Å, and with *U*_iso_(H) = 1.2(1.5 for methyl and hydroxyl)*U*_eq_(C,O).

### Crystal data

**Table:**

[Cd_2_I_4_(C_12_H_12_N_2_O_2_)_2_]	*F*(000) = 1072
*M**_r_* = 1164.89	*D*_x_ = 2.489 Mg m^−^^3^
Monoclinic, *P*2_1_/*c*	Mo *K*α radiation, λ = 0.71073 Å
Hall symbol: -P 2ybc	Cell parameters from 998 reflections
*a* = 9.6684 (6) Å	θ = 2.2–29.3°
*b* = 10.1083 (7) Å	µ = 5.38 mm^−^^1^
*c* = 16.4970 (13) Å	*T* = 298 K
β = 105.365 (5)°	Needle, colorless
*V* = 1554.64 (19) Å^3^	0.33 × 0.09 × 0.05 mm
*Z* = 2	

### Data collection

**Table:**

Bruker APEX CCD diffractometer	4186 independent reflections
Radiation source: fine-focus sealed tube	3589 reflections with *I* > 2σ(*I*)
graphite	*R*_int_ = 0.037
φ and ω scans	θ_max_ = 29.3°, θ_min_ = 2.2°
Absorption correction: multi-scan (*SADABS*; Sheldrick, 1996)	*h* = −13→13
*T*_min_ = 0.560, *T*_max_ = 0.760	*k* = −13→13
12218 measured reflections	*l* = −22→22

### Refinement

**Table:**

Refinement on *F*^2^	Primary atom site location: structure-invariant direct methods
Least-squares matrix: full	Secondary atom site location: difference Fourier map
*R*[*F*^2^ > 2σ(*F*^2^)] = 0.027	Hydrogen site location: inferred from neighbouring sites
*wR*(*F*^2^) = 0.056	H-atom parameters constrained
*S* = 1.08	*w* = 1/[σ^2^(*F*_o_^2^) + (0.0193*P*)^2^ + 1.4317*P*] where *P* = (*F*_o_^2^ + 2*F*_c_^2^)/3
4186 reflections	(Δ/σ)_max_ = 0.001
174 parameters	Δρ_max_ = 0.61 e Å^−^^3^
0 restraints	Δρ_min_ = −0.69 e Å^−^^3^

### Fractional atomic coordinates and isotropic or equivalent isotropic displacement parameters (Å^2^)

**Table:**

	*x*	*y*	*z*	*U*_iso_*/*U*_eq_	
C1	0.1500 (4)	0.9278 (4)	−0.0544 (2)	0.0400 (8)	
H1	0.2045	0.9914	−0.0726	0.048*	
C2	0.0103 (4)	0.9080 (4)	−0.1006 (2)	0.0449 (9)	
H2	−0.0289	0.9575	−0.1488	0.054*	
C3	−0.0696 (4)	0.8134 (5)	−0.0738 (2)	0.0487 (10)	
H3	−0.1637	0.7970	−0.1042	0.058*	
C4	−0.0093 (4)	0.7425 (4)	−0.0012 (2)	0.0429 (8)	
H4	−0.0622	0.6790	0.0182	0.051*	
C5	0.1307 (3)	0.7683 (3)	0.0414 (2)	0.0321 (6)	
C6	0.2084 (3)	0.6988 (3)	0.1231 (2)	0.0333 (7)	
C7	0.3574 (5)	0.5630 (4)	0.0613 (3)	0.0546 (10)	
H7A	0.3376	0.4787	0.0825	0.082*	
H7B	0.4517	0.5619	0.0525	0.082*	
H7C	0.2878	0.5808	0.0090	0.082*	
C8	0.2258 (3)	0.7955 (3)	0.1957 (2)	0.0328 (7)	
C9	0.1399 (4)	0.7896 (4)	0.2505 (2)	0.0425 (8)	
H9	0.0728	0.7222	0.2462	0.051*	
C10	0.1556 (4)	0.8853 (5)	0.3119 (2)	0.0499 (10)	
H10	0.0983	0.8836	0.3492	0.060*	
C11	0.2557 (5)	0.9825 (4)	0.3176 (2)	0.0469 (9)	
H11	0.2684	1.0473	0.3590	0.056*	
C12	0.3383 (4)	0.9825 (4)	0.2602 (2)	0.0409 (8)	
H12	0.4066	1.0487	0.2640	0.049*	
N1	0.2106 (3)	0.8594 (3)	0.01554 (17)	0.0330 (6)	
N2	0.3235 (3)	0.8920 (3)	0.20032 (18)	0.0340 (6)	
O1	0.3501 (2)	0.6642 (2)	0.12090 (16)	0.0373 (5)	
O2	0.1277 (3)	0.5886 (3)	0.13179 (18)	0.0478 (6)	
H2A	0.1654	0.5513	0.1763	0.072*	
Cd1	0.45074 (2)	0.89825 (2)	0.095665 (15)	0.03330 (6)	
I1	0.46360 (3)	1.17684 (2)	0.059856 (16)	0.03945 (6)	
I2	0.71540 (3)	0.85526 (3)	0.213660 (16)	0.04384 (7)	

### Atomic displacement parameters (Å^2^)

**Table:**

	*U*^11^	*U*^22^	*U*^33^	*U*^12^	*U*^13^	*U*^23^
C1	0.0416 (18)	0.044 (2)	0.0351 (17)	0.0046 (16)	0.0115 (14)	0.0072 (15)
C2	0.0449 (19)	0.057 (2)	0.0317 (17)	0.0187 (18)	0.0077 (15)	0.0028 (16)
C3	0.0281 (16)	0.075 (3)	0.0395 (19)	0.0080 (18)	0.0027 (14)	−0.0097 (19)
C4	0.0319 (16)	0.054 (2)	0.0418 (18)	−0.0047 (16)	0.0080 (14)	−0.0011 (17)
C5	0.0293 (15)	0.0334 (16)	0.0330 (15)	0.0016 (13)	0.0071 (12)	−0.0014 (13)
C6	0.0292 (14)	0.0314 (16)	0.0383 (17)	−0.0022 (13)	0.0073 (13)	0.0057 (14)
C7	0.061 (2)	0.040 (2)	0.061 (3)	0.0116 (19)	0.013 (2)	−0.0058 (19)
C8	0.0308 (15)	0.0351 (17)	0.0314 (15)	−0.0013 (13)	0.0062 (12)	0.0049 (13)
C9	0.0420 (18)	0.048 (2)	0.0395 (18)	−0.0023 (17)	0.0146 (15)	0.0121 (17)
C10	0.051 (2)	0.063 (3)	0.041 (2)	0.002 (2)	0.0227 (17)	0.0076 (19)
C11	0.061 (2)	0.048 (2)	0.0345 (18)	0.0048 (19)	0.0166 (17)	0.0008 (16)
C12	0.0429 (19)	0.0398 (19)	0.0408 (18)	−0.0067 (16)	0.0127 (15)	−0.0038 (16)
N1	0.0308 (13)	0.0369 (14)	0.0311 (13)	0.0025 (12)	0.0080 (11)	0.0040 (12)
N2	0.0334 (13)	0.0354 (15)	0.0344 (14)	−0.0026 (11)	0.0109 (11)	0.0015 (12)
O1	0.0343 (12)	0.0350 (12)	0.0400 (13)	0.0055 (10)	0.0054 (10)	0.0005 (10)
O2	0.0493 (15)	0.0385 (14)	0.0524 (16)	−0.0161 (12)	0.0080 (12)	0.0098 (12)
Cd1	0.02758 (11)	0.03613 (13)	0.03617 (12)	−0.00235 (9)	0.00842 (9)	0.00239 (10)
I1	0.04378 (12)	0.03060 (11)	0.04923 (13)	−0.00043 (9)	0.02155 (10)	−0.00408 (9)
I2	0.03782 (12)	0.04460 (14)	0.04293 (13)	0.00002 (10)	−0.00012 (9)	0.00529 (10)

### Geometric parameters (Å, °)

**Table:**

C1—N1	1.340 (4)	C8—N2	1.346 (4)
C1—C2	1.378 (5)	C8—C9	1.383 (5)
C1—H1	0.9300	C9—C10	1.380 (6)
C2—C3	1.375 (6)	C9—H9	0.9300
C2—H2	0.9300	C10—C11	1.365 (6)
C3—C4	1.384 (6)	C10—H10	0.9300
C3—H3	0.9300	C11—C12	1.391 (5)
C4—C5	1.375 (5)	C11—H11	0.9300
C4—H4	0.9300	C12—N2	1.327 (4)
C5—N1	1.342 (4)	C12—H12	0.9300
C5—C6	1.529 (5)	Cd1—N1	2.381 (3)
C6—O2	1.389 (4)	Cd1—N2	2.371 (3)
C6—O1	1.424 (4)	Cd1—O1	2.633 (2)
C6—C8	1.520 (5)	O2—H2A	0.8200
C7—O1	1.433 (5)	Cd1—I1	2.8868 (4)
C7—H7A	0.9600	Cd1—I1^i^	2.9951 (4)
C7—H7B	0.9600	Cd1—I2	2.8082 (4)
C7—H7C	0.9600		
			
N1—C1—C2	122.8 (4)	C11—C10—H10	120.2
N1—C1—H1	118.6	C9—C10—H10	120.2
C2—C1—H1	118.6	C10—C11—C12	118.5 (4)
C3—C2—C1	118.4 (3)	C10—C11—H11	120.7
C3—C2—H2	120.8	C12—C11—H11	120.7
C1—C2—H2	120.8	N2—C12—C11	122.6 (3)
C2—C3—C4	119.7 (3)	N2—C12—H12	118.7
C2—C3—H3	120.2	C11—C12—H12	118.7
C4—C3—H3	120.2	C1—N1—C5	118.1 (3)
C5—C4—C3	118.5 (4)	C1—N1—Cd1	122.2 (2)
C5—C4—H4	120.8	C5—N1—Cd1	119.7 (2)
C3—C4—H4	120.8	C12—N2—C8	118.6 (3)
N1—C5—C4	122.6 (3)	C12—N2—Cd1	123.3 (2)
N1—C5—C6	114.0 (3)	C8—N2—Cd1	118.0 (2)
C4—C5—C6	123.5 (3)	C6—O1—C7	114.5 (3)
O2—C6—O1	112.1 (3)	C6—O1—Cd1	100.48 (18)
O2—C6—C8	112.5 (3)	C7—O1—Cd1	116.5 (2)
O1—C6—C8	105.7 (3)	C6—O2—H2A	109.5
O2—C6—C5	107.5 (3)	N2—Cd1—N1	77.41 (9)
O1—C6—C5	110.0 (3)	N2—Cd1—O1	64.93 (9)
C8—C6—C5	108.9 (3)	N1—Cd1—O1	65.95 (8)
O1—C7—H7A	109.5	N2—Cd1—I2	92.54 (7)
O1—C7—H7B	109.5	N1—Cd1—I2	159.22 (7)
H7A—C7—H7B	109.5	O1—Cd1—I2	93.35 (5)
O1—C7—H7C	109.5	N2—Cd1—I1	103.52 (7)
H7A—C7—H7C	109.5	N1—Cd1—I1	97.81 (7)
H7B—C7—H7C	109.5	O1—Cd1—I1	161.21 (5)
N2—C8—C9	121.9 (3)	I2—Cd1—I1	102.242 (11)
N2—C8—C6	116.2 (3)	N2—Cd1—I1^i^	158.66 (7)
C9—C8—C6	121.8 (3)	N1—Cd1—I1^i^	86.16 (7)
C10—C9—C8	118.8 (4)	O1—Cd1—I1^i^	96.05 (5)
C10—C9—H9	120.6	I2—Cd1—I1^i^	98.429 (11)
C8—C9—H9	120.6	I1—Cd1—I1^i^	92.019 (9)
C11—C10—C9	119.6 (3)	Cd1—I1—Cd1^i^	87.981 (9)
			
N1—C1—C2—C3	−0.4 (6)	C8—C6—O1—Cd1	−60.1 (2)
C1—C2—C3—C4	1.0 (6)	C5—C6—O1—Cd1	57.4 (3)
C2—C3—C4—C5	−0.9 (6)	C12—N2—Cd1—N1	−131.9 (3)
C3—C4—C5—N1	0.2 (5)	C8—N2—Cd1—N1	44.4 (2)
C3—C4—C5—C6	178.8 (3)	C12—N2—Cd1—O1	159.0 (3)
N1—C5—C6—O2	−166.4 (3)	C8—N2—Cd1—O1	−24.7 (2)
C4—C5—C6—O2	14.9 (5)	C12—N2—Cd1—I2	66.5 (3)
N1—C5—C6—O1	−44.1 (4)	C8—N2—Cd1—I2	−117.2 (2)
C4—C5—C6—O1	137.2 (3)	C12—N2—Cd1—I1	−36.7 (3)
N1—C5—C6—C8	71.4 (3)	C8—N2—Cd1—I1	139.5 (2)
C4—C5—C6—C8	−107.3 (4)	C12—N2—Cd1—I1^i^	−172.4 (2)
O2—C6—C8—N2	168.1 (3)	C8—N2—Cd1—I1^i^	3.9 (4)
O1—C6—C8—N2	45.4 (4)	C1—N1—Cd1—N2	133.1 (3)
C5—C6—C8—N2	−72.7 (4)	C5—N1—Cd1—N2	−45.4 (2)
O2—C6—C8—C9	−15.8 (4)	C1—N1—Cd1—O1	−159.0 (3)
O1—C6—C8—C9	−138.5 (3)	C5—N1—Cd1—O1	22.5 (2)
C5—C6—C8—C9	103.4 (4)	C1—N1—Cd1—I2	−164.33 (19)
N2—C8—C9—C10	−0.2 (5)	C5—N1—Cd1—I2	17.2 (4)
C6—C8—C9—C10	−176.1 (3)	C1—N1—Cd1—I1	30.9 (3)
C8—C9—C10—C11	−0.6 (6)	C5—N1—Cd1—I1	−147.6 (2)
C9—C10—C11—C12	0.7 (6)	C1—N1—Cd1—I1^i^	−60.6 (3)
C10—C11—C12—N2	0.0 (6)	C5—N1—Cd1—I1^i^	120.9 (2)
C2—C1—N1—C5	−0.3 (5)	C6—O1—Cd1—N2	45.57 (19)
C2—C1—N1—Cd1	−178.8 (3)	C7—O1—Cd1—N2	169.9 (3)
C4—C5—N1—C1	0.4 (5)	C6—O1—Cd1—N1	−41.29 (19)
C6—C5—N1—C1	−178.3 (3)	C7—O1—Cd1—N1	83.0 (2)
C4—C5—N1—Cd1	179.0 (3)	C6—O1—Cd1—I2	136.82 (18)
C6—C5—N1—Cd1	0.3 (4)	C7—O1—Cd1—I2	−98.9 (2)
C11—C12—N2—C8	−0.8 (5)	C6—O1—Cd1—I1	−9.4 (3)
C11—C12—N2—Cd1	175.4 (3)	C7—O1—Cd1—I1	114.9 (3)
C9—C8—N2—C12	0.9 (5)	C6—O1—Cd1—I1^i^	−124.33 (18)
C6—C8—N2—C12	177.0 (3)	C7—O1—Cd1—I1^i^	0.0 (2)
C9—C8—N2—Cd1	−175.5 (3)	N2—Cd1—I1—Cd1^i^	−165.25 (7)
C6—C8—N2—Cd1	0.5 (4)	N1—Cd1—I1—Cd1^i^	−86.40 (7)
O2—C6—O1—C7	51.3 (4)	O1—Cd1—I1—Cd1^i^	−115.52 (17)
C8—C6—O1—C7	174.3 (3)	I2—Cd1—I1—Cd1^i^	99.076 (12)
C5—C6—O1—C7	−68.3 (4)	I1^i^—Cd1—I1—Cd1^i^	0.0
O2—C6—O1—Cd1	176.9 (2)		

Symmetry codes: (i) −*x*+1, −*y*+2, −*z*.

### Hydrogen-bond geometry (Å, °)

**Table:**

*D*—H···*A*	*D*—H	H···*A*	*D*···*A*	*D*—H···*A*
O2—H2A···I2^ii^	0.82	2.73	3.509 (3)	160

Symmetry codes: (ii) −*x*+1, *y*−1/2, −*z*+1/2.
